# Remdesivir in Patients With Severe Kidney Dysfunction: A Secondary Analysis of the CATCO Randomized Trial

**DOI:** 10.1001/jamanetworkopen.2022.29236

**Published:** 2022-08-29

**Authors:** Matthew Cheng, Rob Fowler, Srinivas Murthy, Ruxandra Pinto, Nancy L. Sheehan, Alice Tseng

**Affiliations:** 1 McGill University Health Centre, Montréal, Québec, Canada; 2 Sunnybrook Health Sciences Centre, Toronto, Ontario, Canada; 3 Faculty of Medicine, University of British Columbia, Vancouver, British Columbia, Canada; 4 Faculty of Pharmacy, University of Montréal, Montréal, Québec, Canada; 5 University Health Network, Toronto General Hospital, Toronto, Ontario, Canada

## Abstract

This secondary analysis of a randomized clinical trial examines the risk of kidney or hepatic toxic effects among patients with impaired kidney function at baseline treated with remdesivir.

## Introduction

Remdesivir is a broad-spectrum antiviral that reduces hospitalization and may decrease mortality among noncritically ill inpatients with COVID-19.^1,2^ Remdesivir is not recommended for use in patients with an estimated glomerular filtration rate (eGFR) less than 30 mL/min1.73 m^2^ owing to the presence of excipients^3^ that may accumulate in kidney dysfunction and worsen kidney or hepatic outcomes.^4^

The Canadian Treatments for COVID-19 (CATCO) trial randomized patients to remdesivir or standard care between August 1, 2020, and April 1, 2021, as part of the global Solidarity trial.^5^ CATCO did not have kidney function–based exclusion criteria. We report on patients with impaired kidney function at baseline as a post hoc analysis to examine the risk of kidney or hepatic toxic effects with remdesivir administration.

## Methods

Details of CATCO are presented elsewhere.^5^ This study was approved by all ethics committees and followed the CONSORT reporting guideline. Patients with impaired kidney function (eGFR <30 mL/min/1.73 m^2^ at randomization)^6^ received open-label remdesivir or best-quality supportive care (eFigure in Supplement 1). The trial protocol is available in Supplement 2. Lyophilized remdesivir was diluted and administered intravenously with a loading dose of 200 mg on day 1, followed by daily 100-mg doses for 9 days or until discharge. There was no dose adjustment for baseline kidney dysfunction or dialysis. Adverse events were reported by clinical staff.

We report all-cause mortality (unadjusted and adjusted for sex and baseline eGFR), adverse events, incidence of new ventilation or hemodialysis as proportions, and outcomes of day 5 eGFR and alanine aminotransferase levels, using medians (IQRs). There were no a priori levels of significance established. Given baseline imbalance, analysis of covariance was performed for the change in eGFR, adjusting for baseline eGFR. As sensitivity analysis, we report the results of binary outcomes for all patients in the main trial with eGFR 60 mL/min/1.73 m^2^, with 2-sided *P* values obtained through χ^2^ tests. All analyses were performed in SAS, version 9.4 (SAS Institute Inc).

## Results

Of 1281 patients, 59 (remdesivir, 34; standard care, 25) had a baseline eGFR less than 30 mL/min/1.73 m^2^; median age was 74 (IQR, 63-86) years (Table 1). There were imbalances between the groups, with more men and a lower median eGFR at baseline in the standard-care group. At baseline, few patients were undergoing hemodialysis; median eGFR was 18.9 (IQR, 10.2-24.2) mL/min/1.73 m^2^ across groups.

**Table 1.** Baseline Characteristics of Randomized Patients With eGFR Less Than 30 mL/min/1.73 m^2^ at Time of Randomization

Variable^a^	No. (%)		
	Overall (N = 59)	Remdesivir (n = 34)	Standard care (n = 25)
Age, median (IQR), y	74 (63-86)	74 (66-86)	80 (63-85)
Sex at birth			
Female	29 (49.2)	21 (61.8)	8 (32.0)
Male	30 (50.8)	13 (38.2)	17 (68.0)
Clinical frailty score, median (IQR)^b^	5 (4-6)	4 (3-6)	5 (4-6)
Baseline severity			
In ICU	18 (30.5)	11 (32.4)	7 (28.0)
No oxygen	4 (6.8)	4 (11.8)	0
Receiving oxygen	30 (50.8)	16 (47.1)	14 (56.0)
High-flow nasal cannulae	14 (23.7)	6 (17.6)	8 (32.0)
Noninvasive ventilation	2 (3.4)	1 (2.9)	1 (4.0)
Invasive ventilation	9 (15.3)	7 (20.6)	2 (8.0)
Baseline creatinine, median (IQR), mg/dL	2.97 (2.22-5.54)	2.62 (2.02-5.17)	3.88 (2.65-6.1)
Baseline eGFR, median (IQR), mL/min/1.73 m^2^	18.9 (10.2-24.2)	22.7 (10.5-26.6)	12.4 (8.8-20.6)
Baseline need for dialysis	15 (25.4)	9 (26.5)	6 (24.0)
Baseline ALT, median (IQR), U/L^c^	28 (21-45)	27 (21-49)	35 (21-43)

Abbreviations: ALT, alanine aminotransferase; eGFR, estimated glomerular filtration rate; ICU, intensive care unit.

SI conversion: To convert ALT to microkatals per liter, multiply by 0.0167; creatinine to micromoles per liter, 88.4.

^a^ Data on race and ethnicity were collected but are not reported owing to the small numbers.

^b^ Clinical Frailty score is a 9-point scoring system, with lower scores indicating a lower level of frailty.

^c^ Available in 39 patients.

The median duration of remdesivir administered in the intervention arm was 10 (IQR, 6-10) days (mean, 8.2 days). There were no significant differences in key outcomes between groups (Table 2). Adjustment for sex and baseline eGFR did not change mortality outcomes (odds ratio, 0.74; 95% CI, 0.23-2.40). Analysis of covariance for change in eGFR showed no significant difference between groups. For patients with eGFR less than 60 mL/min/1.73 m^2^ (n = 248) at randomization, there was no significant difference between those receiving remdesivir (n = 122) or standard care (n = 126) in hospital mortality (35.2% vs 42.1%; *P* = .26), new mechanical ventilation (10.6% vs 15.7%; *P* = .27), or new dialysis (6.2% vs 5.0%; *P* = .70).

**Table 2.** Clinical Outcomes of Patients With eGFR Less Than 30 mL/min/1.73 m^2^ at Time of Randomization^a^

Variable	Remdesivir (n = 34)	Standard care (n = 25)	Difference in means (95% CI)
Hospital death, No. (%), unadjusted	13 (40.6)	13 (52.0)	RR, 0.78 (0.41 to 1.49)
New mechanical ventilation in those not ventilated at baseline, No. (%)	4 (14.8)	6 (26.1)	RR, 0.57 (0.15 to 1.80)
Total length of stay, d			
Mean (SD)	23.1 (20.5)	21.6 (28.8)	1.5 (–11.5 to 14.6)
Median (IQR)	16.5 (10-29.5)	11 (6-25)	NA
Day 5 creatinine, mg/dL^b^			
Mean (SD)	2.83 (2.15)	4.12 (2.41)	–1.29 (–2.66 to 0.09)
Median (IQR)	1.80 (1.32-3.40)	3.47 (2.02-5.99)	NA
Day 5 eGFR, mL/min/1.73 m^2^^b^			
Mean (SD)	31.2 (19.2)	20.5 (13.9)	10.7 (0.20 to 21.2)
Median (IQR)	29.2 (14.2-45)	16.5 (8.5-30.9)	NA
ANCOVA for change in eGFR			–0.26 (–7.95 to 7.42)
Day 5 ALT^c^			
Mean (SD)	40.8 (36.4)	91.9 (187)	–51.1 (–177.2 to 75.0)
Median (IQR)	32.5 (15-47)	31 (22-52)	NA
New dialysis in those not receiving dialysis at baseline, No. (%)	5 (20.0)	4 (21.1)	RR, 0.95 (0.25 to 3.56)
Any adverse event, No. (%)	3 (8.8)	6 (24.0)	RR, 0.37 (0.05 to 1.33)

Abbreviations: ALT, alanine aminotransferase; ANCOVA, analysis of covariance; eGFR, estimated glomerular filtration rate; NA, not applicable; RR, relative risk.

SI conversion: To convert ALT to microkatals per liter, multiply by 0.0167; creatinine to micromoles per liter, 88.4.

^a^ Percentages reported for those with available outcome.

^b^ Available in 45 patients.

^c^ Available in 31 patients.

## Discussion

In patients with eGFR less than 30 mL/min/1.73 m^2^ at baseline who received remdesivir, there was no increased risk of transaminitis or toxic kidney effects at day 5. There was also no significant difference in the need for invasive mechanical ventilation or new dialysis, or mortality. This study is limited by small numbers and baseline imbalance between groups. These findings suggest that remdesivir can be safely administered in patients with kidney dysfunction, balancing possible risks and benefits. The need for assessing kidney function in the absence of clinical suspicion before and during outpatient administration of remdesivir can be questioned.

## Notes

Supplement 1. **eFigure.** Randomization of Participants

Supplement 2.
Trial Protocol

Supplement 3.
Data Sharing Statement
