# Supplementary material for: Remdesivir in Patients With Severe Kidney Dysfunction: A Secondary Analysis of the CATCO Randomized Trial
Source: JAMA Netw Open. 2022 Aug 29;5(8):e2229236. doi: 10.1001/jamanetworkopen.2022.29236 (PMC9425145; doi:10.1001/jamanetworkopen.2022.29236)
Supplement: Supplement 1. — eFigure. Randomization of Participants [file jamanetwopen-e2229236-s001.pdf]

Supplementary Online Content

Cheng M, Fowler R, Murthy S, Pinto R, Sheehan NL, Tseng A. Remdesivir in patients with severe kidney dysfunction: a secondary analysis of the CATCO randomized trial. *JAMA Netw Open*. 2022;5(8):e2229236. doi:10.1001/jamanetworkopen.2022.29236

eFigure. Randomization of Participants

This supplementary material has been provided by the authors to give readers additional information about their work.

© 2022 Cheng M et al. *JAMA Network Open.*

eFigure. Randomization of Participants

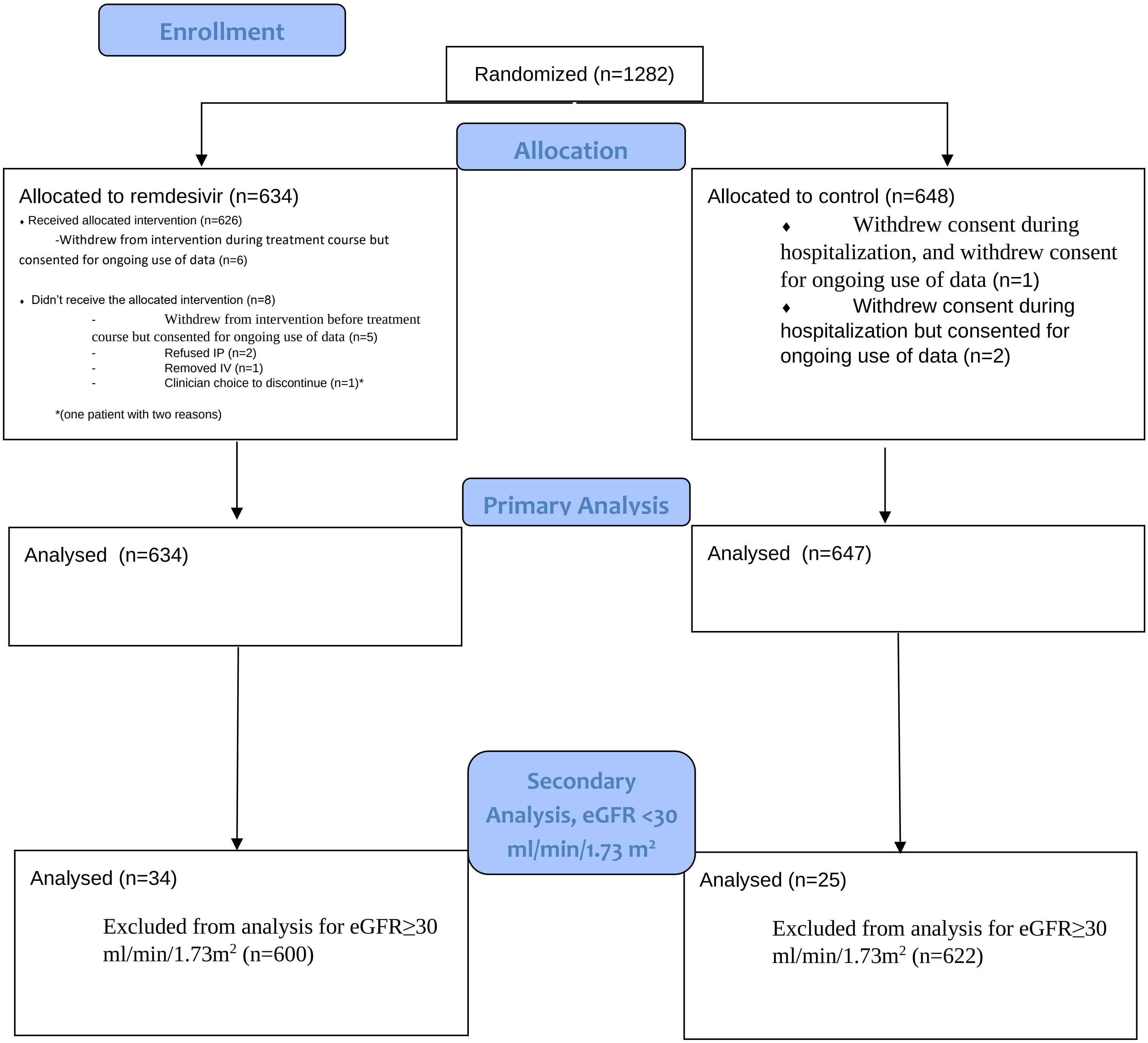

© 2022 Cheng M et al. *JAMA Network Open.*
