# Supplementary material for: Remdesivir in Patients With Severe Kidney Dysfunction: A Secondary Analysis of the CATCO Randomized Trial
Source: JAMA Netw Open. 2022 Aug 29;5(8):e2229236. doi: 10.1001/jamanetworkopen.2022.29236 (PMC9425145; doi:10.1001/jamanetworkopen.2022.29236)
Supplement: Supplement 2. — Trial Protocol [file jamanetwopen-e2229236-s002.pdf]

(CATCO)

A Multi-centre, Adaptive, Randomized, Open-label, Controlled Clinical Trial of the Safety and Efficacy of Investigational Therapeutics for the Treatment of COVID-19 in Hospitalized Patients

[in conjunction with the Public health emergency SOLIDARITY trial (World Health Organization)]

Protocol Number: *2114*

Confidential

National Principal Investigator: Dr. Srinivas Murthy
Provincial Principal Investigator: Dr. Robert Fowler
Regulatory Sponsor: Sunnybrook Research Institute
Funding Agency: Canadian Institutes of Health Research
Investigational Products: Remdesivir
Interferon-beta-1a

Coordinating Centre: Centre for Clinical Trial Support (CCTS)
Sunnybrook Research Institute
C8-2075 Bayview Avenue
Toronto, ON M4N 3M5
Canada
Phone: (416) 480-5632
Email: *CATCO@sunnybrook.ca*.

For Peer Review Only

SPONSOR STATEMENT OF COMPLIANCE

This study will comply with the International Council for Harmonisation of Technical Requirements for Pharmaceuticals for Human use Good Clinical Practice E6 (ICH-GCP), World Medical Association Declaration of Helsinki: Ethical Principles for Medical Research Involving Human Participants, as well as applicable regulatory and institutional requirements.

Personnel listed below are authorized to sign the protocol and any subsequent protocol amendments on behalf of the sponsor:

Name:<br>*(Print)*	Robert Fowler
Title:<br>*(Print)*	Physician & Senior Scientist, Sunnybrook HSC
Signature:	
Date of Approval:<br>*(yyyy-mmm-dd)*	2020-Sep-10

Confidential

For Peer Review Only

PROTOCOL SIGNATURE PAGE

I have read this protocol in its entirety and its appendices. I agree to comply with the requirements of the study protocol and procedures for data recording/reporting and acknowledge my responsibility for the well-being of each research participant, and to ensure that all persons involved in study activities are adequately informed about the protocol, the investigational product, and their trial-related duties. The signature below constitutes the agreement to conduct this study in accordance with the REB approved protocol, GCP and applicable regulatory requirements, including confidentiality, ethical guidelines and regulations regarding the conduct of research in humans.

Qualified Investigator:

Name:
(Print) ____________________________________

Title & Institution:
(Print) ____________________________________

Signature: ____________________________________

Date of signature:
(yyyy-mmm-dd) ____________________________________

Confidential

For Peer Review Only

TABLE OF CONTENTS

PAGE
SPONSOR STATEMENT OF COMPLIANCE 1
PROTOCOL SIGNATURE PAGE 2
TABLE OF CONTENTS 3
LIST OF ABBREVIATIONS 6
PROTOCOL SUMMARY 8
1 KEY ROLES AND CONTACT INFORMATION 10
2 INTRODUCTION 11
2.1 Background 12
2.2 Preclinical Data 13
2.3 Clinical Data to Date 13
2.4 Potential Risks/Benefits and Rationale 13
2.4.1 Dose Selection 14
3 STUDY OBJECTIVES 15
3.1 Primary Objective 15
3.2 Secondary Objective 15
3.3 Exploratory Objective(s) 15
4 STUDY DESIGN 16
4.1 General Design 16
4.2 Primary Outcomes/Endpoint(s) 16
4.3 Secondary Outcomes/Endpoint(s) 16
4.3.1 Clinical Severity 16
4.3.2 Hospitalization 17
4.3.3 Mortality 18
4.3.4 Safety 18
4.4 Exploratory Outcome 18
5 PARTICIPANT SELECTION & WITHDRAWAL 19
5.1 Inclusion Criteria 19
5.2 Exclusion Criteria 19
5.3 Participant Recruitment 19
5.3.1 Randomization Procedures 20
5.3.2 Blinding and Unblinding Procedures 20
5.4 Participant Withdrawal and Discontinuation of IP 20
5.4.1 Reasons for Withdrawal/Discontinuation of IP 20
5.4.2 Data Collection and Follow-up for Withdrawn Participants 21
6 INTERVENTIONS 22
6.1 Investigational Product 22
6.1.1 Acquisition, Formulation and Packaging 22
6.1.2 Treatment Assignment Procedures 23
6.1.3 Dosage, Preparation and Administration 23
6.1.4 Dose Modification 24
6.1.5 Accountability: Receiving, Storage, Dispensing and Return 24

Confidential

For Peer Review Only

6.1.6 Prior and Concomitant Medications/Treatments 25
6.1.7 Safety Considerations 25
7 STUDY SCHEDULE AND PROCEDURES 27
7.1 Screening 27
7.2 Baseline/Enrollment (Study Day 1) 27
7.3 Study visits and Follow up 28
7.3.1 Study Day 2 (+/-1) through Discharge 28
7.3.2 Study Days 15 (+/-2), 29 (+/-3) and 60 (+/-3) 29
7.3.3 Follow-Up in Pregnant Participants 29
7.4 Protocol Deviations 29
8 ASSESSMENT OF SAFETY 31
8.1 Definitions 31
8.1.1 Adverse Events 31
8.1.2 Adverse Events of Special Interest 32
8.1.3 Serious Adverse Events 32
8.1.4 Unexpected Adverse Event 32
8.1.5 Unexpected Adverse Drug Reaction (ADR) 32
8.2 Assessment of an Adverse Event 33
8.2.1 Relationship (Causality/Relatedness) 33
8.2.2 Expectedness 33
8.2.3 Seriousness 34
8.2.4 Severity 34
8.3 Adverse Event Recording 34
8.4 Reporting of SAEs and Unanticipated Events 35
8.4.1 Investigator reporting: Notifying the REB 36
8.4.2 Investigator reporting: Notifying the Sponsor 36
8.4.3 Investigator Reporting to Subjects 36
8.4.4 Sponsor Reporting of SUADRs: Notifying Health Canada 36
8.4.5 Sponsor Reporting of SUADRs: Notifying Sites 37
8.4.6 Sponsor Reporting of AEs: Notifying Abbvie and EMD Serono/Merck KGaA, Darmstadt, Germany 37
8.5 Type and Duration of Follow-up for Adverse Events 37
8.6 Reporting and Entry Timelines 37
8.7 Data and Safety Monitoring Board (DSMB) 37
9 SITE MONITORING, AUDITING AND INSPECTING 39
9.1 Site Monitoring Plan 39
9.2 Auditing and Inspecting 39
10 STATISTICAL CONSIDERATIONS 40
10.1 Study Hypotheses 40
10.2 Sample Size Considerations 40
10.3 Planned Interim Analyses 40
10.3.1 Safety Review 40
10.3.2 Efficacy Review 41
10.4 Stopping Rules 41

Confidential

For Peer Review Only

10.5 Final Analysis Plan 41
10.5.1 Primary Efficacy Endpoint 42
10.5.2 Secondary Endpoint 42
10.5.3 Safety Analysis 42
10.5.4 Sub-Group Analysis 42
10.5.5 Exploratory Analysis 43
11 DATA HANDLING AND RECORD KEEPING 44
11.1 Confidentiality 44
11.2 Source Documents 44
11.3 Data Management Responsibilities 45
11.4 Data Capture 45
11.4.1 Case Report Forms 46
11.5 Records Retention 46
11.6 Clinical Trial Registration 47
12 QUALITY CONTROL AND QUALITY ASSURANCE 48
13 ETHICS CONSIDERATIONS 49
13.1 Ethical Standard 49
13.2 Research Ethics Board (REB) 49
13.3 Consent 49
13.4 Secondary Use of Stored Data 50
14 PUBLICATION/DATA SHARING POLICY 51
14.1 Data Sharing 51
14.1.1 Data Sharing for Secondary Research 51
14.2 Conflict of Interest 51
15 LITERATURE REFERENCES 52
APPENDIX A: SCHEDULE OF EVENTS 54

Confidential

For Peer Review Only

LIST OF ABBREVIATIONS

The following abbreviations describe terms, documents and study personnel used in the conduct of this study protocol.

AE	Adverse Event
ALT	Alanine Transaminases
AST	Aspartate Transaminase
BEEC	Blinded Endpoint Evaluation Committee
BP	Blood Pressure
CCTS	Centre for Clinical Trial Support
CFR	Code of Federal Regulations
CI	Confidence Interval
CLIA	Clinical Laboratory Improvement Amendments
CMP	Clinical Monitoring Plan
CMS	Clinical Material Services
Cr	Creatinine
CRF	Case Report Form
DSMB	Data Safety Monitoring Board
EDC	Electronic Data Capture
GCP	Good Clinical Practice
GLP	Good Laboratory Practices
Hgb	Haemoglobin
HR	Heart Rate
IB	Investigator's Brochure
IP	Investigational Product
ICD	International Classification of Diseases
ICF	Informed Consent Form
ICH	International Council for Harmonisation of Technical Requirements for Pharmaceuticals for Human Use
ISF	Investigator Site File
MCG	Microgram
MedDRA	Medical Dictionary for Regulatory Activities
MERS	Middle East Respiratory Syndrome
MOP	Manual of Procedures
N	Number (typically refers to subjects)
NDA	New Drug Application
OP	Oropharyngeal swabbing
PHI	Protected Health Information
PI	Principal Investigator
PLT	Platelet
PM	Product Monograph
PP	Per Protocol
PT	Prothrombin Time
QI	Qualified Investigator
RCB	Randomized Control Trial
REB	Research Ethics Board
SAE	Serious Adverse Event

Confidential

For Peer Review Only

SAP	Statistical Analysis Plan
SARS	Severe Acute Respiratory Syndrome
SDCC	Statistical and Data Coordinating Centre
SDSP	Study Data Standardization Plan
SMC	Safety Monitoring Committee
SNP	Single Nucleotide Polymorphisms
SpO_2	Oxygen saturation
SOE	Schedule of Events
SOP	Standard Operating Procedure
SRI	Sunnybrook Research Institute
SUADR	Suspected Unexpected Adverse Drug Reaction
SUSAR	Suspected Unexpected Serious Adverse Reaction
T. Bili	Total Bilirubin
TMF	Trial Master File
UP	Unanticipated Problem
US	United States
WBC	White Blood Cell

Confidential

PROTOCOL SUMMARY

**Protocol Title (Short Title) and Sub-Title**	A Multi-centre, Adaptive, Randomized, Open-label, Controlled Clinical Trial of the Safety and Efficacy of Investigational Therapeutics for the Treatment of COVID-19 in Hospitalized Patients (CATCO: Canadian Treatments for COVID-19), in conjunction with the Public health emergency SOLIDARITY trial (World Health Organization)
**Protocol Number**	2114
**Phase**	II
**Study Design**	This study is an adaptive, randomized, open-label, controlled clinical trial, in collaboration with countries around the world through the World Health Organization.<br><br>Subjects will be randomized to receive either standard-of-care products or the study medication plus standard of care, while being hospitalized for COVID-19.<br><br>Participants will be randomized to one of the following groups:<br>1. Remdesivir 200mg IV on day 1, followed by 100 mg IV daily infusion for 9 days plus optimized supportive care, OR<br>2. Interferon-beta-1a, 44 micrograms subcutaneously on days 1, 3 and 6 plus optimized supportive care, OR<br>3. Optimized support care<br>all or until discharge from hospital, whichever occurs first<br><br>Subjects will be assessed daily while hospitalized, including Oropharyngeal (OP) / Nasopharyngeal (NP) swabbing at baseline and on days post enrolment 3, 5, 8, 11, 15 and 29 (if still hospitalized, exploratory outcome). Hospitalized subjects will require blood sampling on days 1 and 5. Discharged subjects will be telephoned at Days 15, 29, and 60.
**Study Duration**	2 years
**Setting**	National, multi-centre
**Sample Size**	As a public health emergency that is evolving rapidly, an accurate sample size is impossible to calculate. Interim results will be reviewed at regular intervals by the global DSMB for efficacy.
**Main Inclusion Criteria**	Age ≥ 18 years of age, has laboratory-confirmed SARS-CoV-2 infection as determined by PCR, or other commercial or public health assay in any specimen prior to randomization and is admitted

Confidential

For Peer Review Only

	to hospital at a participating centre.
**Primary Outcome(s):**	All-cause mortality, assessed at hospital discharge.
**Secondary Outcome(s):**	1. To calculate clinical severity using:<br>a. Ordinal Scales to measure: **(1)** the time to improvement of one category from admission, **(2)** subject clinical status at days 3, 5, 8, 11,15, 29, and 60 or discharge, whichever comes later , **(3)** Mean change in the ranking from baseline to day 3, 5, 8, 11, 15, 29, and 60 or discharge, whichever comes later<br>b. Oxygenation**: (1)** oxygen-free days in the first 28 days, **(2)** incidence and duration of new oxygen use during trial<br>c. Mechanical Ventilation: **(1)** ventilator-free days in the first 28 days, **(2)** incidence and duration of new mechanical ventilation use during the trial<br>2. Duration of hospitalization (days) and hospital-free days at day 60;<br>3. 15, 29, and 60-days or discharge, whichever comes later mortality<br>4. Evaluate safety of the intervention during trial as defined by the: cumulative incidence of Grade 3 and 4 AEs and SAEs (using DAIDS 2017)
**Exploratory Outcome(s):**	To evaluate the virologic efficacy of remdesivir and interferon compared to the control arm as assessed by the proportion of subjects with SARS-CoV-2 detectable in OP sample at days 3, 5, 8, 11, 15, and 29.
**Investigational Product and Planned Use**	The study is designed to evaluate remdesivir and interferon as the study interventions, with the possibility of evaluating additional interventions over time.
**Statistical Analysis:**	This is an adaptive, open-label, randomized trial testing a superiority hypothesis without pre-specified stopping rules. Secondary hypotheses have been ordered according to relative importance. These will be described according to the appropriate summary statistics (e.g., proportions for categorical data, means with 95% confidence intervals for continuous data, median for time-to-event data).

Confidential

For Peer Review Only

1 KEY ROLES AND CONTACT INFORMATION

**Regulatory Sponsor:**	Sunnybrook Research Institute (SRI)<br>2075 Bayview Avenue<br>Toronto, ON Canada M4N 3M5
**National Principal Investigator:**	Dr. Srinivas Murthy
**Provincial Principal Investigator:**	Dr. Robert Fowler
**Academic Research Organization:**	Centre for Clinical Trial Support, SRI<br>2075 Bayview Avenue<br>Toronto, ON Canada M4N 3M5
**DSMB**	Independent, to be established.

Confidential

This protocol is adapted from the SOLIDARITY protocol. All elements of the SOLIDARITY protocol are included in this, with added features specific to the Canadian context and to achieve standards for Canadian regulators and ethics committees for oversight and monitoring.

For Peer Review Only

2 INTRODUCTION

In December 2019, the Wuhan Municipal Health Committee identified an outbreak of viral pneumonia cases of unknown cause. Coronavirus RNA was quickly identified in some of these patients. This novel coronavirus has been designated SARS-CoV-2, and the disease caused by this virus has been designated COVID-19. Currently there are no approved therapeutic agents available for coronaviruses.

COVID-19 is a respiratory disease caused by a novel coronavirus (SARS-CoV-2) and causes substantial morbidity and mortality. There is currently no vaccine to prevent COVID-19 or infection with SARS-CoV-2 or therapeutic agent to treat COVID-19. This clinical trial is designed to evaluate potential therapeutics for the treatment of patients hospitalized with COVID-19.

This Protocol is largely based on a series of deliberations of the WHO R&D Blueprint Clinical Trials Expert Group. The Experts include international clinical trials coronavirus experts, regulatory and ethics experts and clinicians, including those treating COVID-19 patients[1]. This version was then further adjusted to facilitate its implementation in Canada.

The global protocol will compare different investigational therapeutic agents to a control arm. Initially this protocol proposed a trial with 2-arms in a 1:1 ratio randomization to either the control arm, consisting of standard of care supportive treatment for COVID-19, or the investigational product, lopinavir/ritonavir plus standard of care, with a subsequent amendment including hydroxychloroquine and remdesivir for a 4-arm randomization in a 1:1:1:1 ratio. Subsequently, the protocol was amended to no longer evaluate hydroxychloroquine and lopinavir/ritonavir, and add interferon into the randomization schema. There will be interim monitoring to allow early stopping for futility, efficacy, or safety and possibly to introduce new therapies as they become available. If one therapy proves to be superior to others in the trial, this treatment will then become the control arm for comparison(s) with new experimental treatment(s).

Because of the possibility that standards of supportive care may vary between trial centres and may also evolve/improve over time as more is learned about successful management of COVID-19, comparisons of safety and efficacy will be based on data from concurrently randomized participants.

An independent Canadian data and safety monitoring board (DSMB) will monitor interim data to make recommendations about early study closure or changes to conduct. This will act in conjunction with a global DSMB, responsible for global efficacy monitoring for accumulated data.

This study utilizes an adaptive design that maximizes efficiency in identifying a safe and efficacious therapeutic agent for COVID-19 during the current outbreak. Some investigational products may be in limited supply and this study design enables continuation of the study even if a product becomes unavailable. In addition, the adaptive design allows for the evaluation of new therapeutic agents as they are identified. As the study will be a multicenter randomized

Confidential

For Peer Review Only

controlled study, it will be possible to acquire rigorous data about the safety and efficacy of investigational therapeutic agents for COVID-19 that will lead to generalizable evidence.

Randomization is essential for establishing efficacy of these new therapeutic agents. Also, collecting clinical and virologic data on enrolled patients using a standardized timeline and collection instruments should provide valuable information about the clinical course of and morbidities associated with severe COVID-19 in a diverse group of hospitalized patients.

This study document is the protocol for research involving human participants. This study is to be conducted according to Canadian and international standards, and in compliance with the protocol, the International Council for Harmonisation of Technical Requirements for Pharmaceuticals for Human Use (ICH) Good Clinical Practice E6 (GCP), World Medical Association Declaration of Helsinki: Ethical Principles for Medical Research Involving Human Participants, as well as applicable regulatory and institutional requirements and research policies.

2.1 Background

Confidential

Coronavirus (CoVs) are positive-sense single stranded enveloped RNA viruses, many of which are commonly found in humans and cause mild symptoms. Over the past two decades, emerging pathogenic CoVs capable of causing life-threatening disease in humans and animals have been identified, namely severe acute respiratory syndrome coronavirus (SARS-CoV) and Middle Eastern respiratory syndrome coronavirus (MERS-CoV).

In December 2019, the Wuhan Municipal Health Committee (Wuhan, China) identified an outbreak of viral pneumonia cases of unknown cause. Coronavirus RNA was quickly identified in some of these patients. This novel coronavirus has been abbreviated as SARS-COV-2 and has 89% nucleotide identity with bat SARS-like-CoVZXC21 and 82% with that of human SARS-CoV[1] There has since been global spread of the virus

This novel coronavirus has been designated SARS-CoV-2, and the disease caused by this virus has been designated COVID-19.

Outbreak forecasting and mathematical modelling suggest that these numbers will continue to rise in many countries over the coming weeks to months.

Global efforts to evaluate novel antivirals and therapeutic strategies to treat COVID-19 have intensified. There is an urgent public health need for rapid development of novel interventions. At present, there is no specific antiviral therapy for coronavirus infections that will improve patient mortality.

For Peer Review Only

2.2 Preclinical Data

Interferon-beta has been shown to have inhibitory effects on coronaviruses, with the beta preparation appearing to have the strongest viral inhibition.[11,12] Through activities directly on the endothelial barrier, there is interferon-dependent upregulation of CD73 expression, leading to a possible hypothesis for benefit in COVID19, where pulmonary endothelial dysfunction is a putative hypothesis.[13]

2.3 Clinical Data to Date

Remdesivir: Remdesivir (GS-5734) is a broad-spectrum nucleotide prodrug that inhibits RNA dependent RNA polymerase activity among a diverse group of RNA viruses including filoviruses (e.g. Ebola, Sudan, Marburg), paramyxoviruses (e.g. RSV, Nipah, Hendra) and pathogenic coronaviruses. It has been tested in Ebola virus disease, with no significant safety signal detected [2]

For COVID19, it has been evaluated in two randomized controlled trials, with a good profile in terms of safety.[3,4] Combined data show that an effectiveness on an outcome of mortality has yet to be shown, with guidelines recommending ongoing randomization (BMJ, in press)

Confidential

Interferon-beta-1a: The antiviral potential of interferon for acute viral infections has long been considered. Humans infected with coronaviruses have been shown to have blockade of the interferon response during critical illness, which has been shown to impair the adaptive immune response. For ARDS, there have been a number of studies examining for benefit, including a large-scale trial of 300 patients.[6] This trial, the INTEREST study, enrolled all patients with ARDS, documenting no difference in day 28 mortality between interferon and placebo (26 and 23%). While not collected, it is presumed that the majority of these patients did not have viral etiology for their ARDS. For COVID19, a randomized trial of interferon, in a combination with lopinavir/ritonavir and ribavirin was shown to be safe, and suggesting further data is required[5]

2.4 Potential Risks/Benefits and Rationale

Remdesivir: Remdesivir has recently been approved for the treatment of COVID-19 in Canada. Remdesivir is a relatively safe therapeutic agent with few subjects experiencing constipation, heartburn, itching, unusual feelings in the ear, dizziness, loss of appetite, nausea, vomiting, shaking of the leg and arm, headache, loose stool, or upset stomach. These adverse events were temporary, lasting only a few days, and none were serious. In randomized studies, no safety signal was observed, with less adverse events than in the placebo group[4]; previous concerns regarding transaminitis were not observed in this well-done placebo-controlled study. There are no significant drug-drug interactions that are expected based on pharmacokinetic data.

Interferon: Interferon has long been used for the treatment of multiple sclerosis and condyloma acuminatum, and is approved for these indications in Canada. Its safety profile is well described, with minimal elevations in liver enzymes reported, and minimal drug-drug

interactions noted in chronic use. Its main side effect profile includes a self-limited flu-like syndrome and mood-swings. In patients with COVID19, a randomized trial reported no increase in adverse events in patients receiving interferon, compared with the control group, with no serious adverse events reported.[5] In critically ill patients with acute respiratory distress syndrome, there was no significant difference in adverse events in a randomized trial of interferon versus placebo[6], with the predominant reported event being fever.

2.4.1 Dose Selection

Remdesivir: The dose of remdesivir used in this study will be the same dose that has been used in the human Ebola clinical trials, with a loading dose of 200 mg on Day 1, followed by 100 mg IV daily for 9 days.

Interferon: The dose of interferon used in this study was chosen based on the dose used for multiple sclerosis, a recent randomized study in critically ill patients with ARDS, the available formulations, and the conversion to intravenous administration.[6] 44 micrograms will be given subcutaneously on days 1, 3 and 6.

Confidential

For Peer Review Only

3 STUDY OBJECTIVES

3.1 Primary Objective

The overall objective of the study is to evaluate the clinical effectiveness of remdesivir or interferon relative to the control arm, in patients hospitalized with COVID-19 in improving mortality.

3.2 Secondary Objective

Evaluate clinical efficacy of, remdesivir or interferon as compared to the control arm.

3.3 Exploratory Objective(s)

To evaluate the virologic efficacy of remdesivir or interferon, as compared to the control arm.

Confidential

4 STUDY DESIGN

4.1 General Design

This study is an adaptive, randomized, open-label, controlled clinical trial.

Subjects will be randomized to receive either standard-of-care products (control) or the study medication plus standard of care while being hospitalized for lab confirmed COVID-19.

Remdesivir will be administered as an intravenous infusion, with a 200 mg IV loading dose on Day 1, followed by 9 days of 100 mg IV daily.

Interferon-beta-1alpha will be administered as a subcutaneous injection of 44 micrograms on days 1, 3, and 6.

Subjects will be assessed daily while hospitalized, including an exploratory outcome of oropharyngeal (OP) / nasopharyngeal swabbing, in centres where capable, on days 1, 3, 5, 8, 11, 15, and 29. Discharged subjects will be telephoned at Days 15, 29, and 60. Hospitalized subjects will require blood sampling on days 1 and 5.

In pregnant individuals, outcomes will be tracked as described in Section 7.3.

4.2 Primary Outcomes/Endpoint(s)

All-cause mortality, assessed at hospital discharge.

4.3 Secondary Outcomes/Endpoint(s)

Secondary outcomes include the evaluation of the clinical efficacy of remdesivir or interferon, or in combination, as compared to the control arm using the following measurements:

4.3.1 Clinical Severity

1. Using an Ordinal scale: (Table 1), the following will be measured:
   a. Time to an improvement of one category from admission
   b. Subject clinical status on an ordinal scale at days 3, 5, 8, 11, 15, 29, and 60 or discharge, whichever comes later, as assessed through a proportional odds model
   c. Mean change in the ranking on an ordinal scale from baseline to days 3, 5, 8, 11, 15, 29, and 60 or discharge, whichever comes later from baseline.

Confidential
For Peer Review Only

Table 1: Ordinal Scale for evaluating subject Clinical Status

Patient State	Descriptor	Score
Uninfected	Uninfected; no viral RNA detected	**0**
Ambulatory	Asymptomatic; viral RNA detected	**1**
	Symptomatic; Independent	**2**
	Symptomatic; Assistance needed	**3**
Hospitalized: Mild disease	Hospitalized; no oxygen therapy	**4**
	Hospitalized; oxygen by mask or nasal prongs	**5**
Hospitalized: Severe disease	Hospitalized; Oxygen by NIV or High flow	**6**
	Intubation & Mechanical ventilation, $pO_2/FIO_2 \geq 150$ or $SpO_2/FIO_2 \geq 200$	**7**
	Mechanical ventilation $pO_2/FIO_2 < 150$ ($SpO_2/FIO_2 < 200$) **or** vasopressors	**8**
	Mechanical ventilation $pO_2/FIO_2 < 150$ **and** vasopressors, dialysis, or ECMO	**9**
Death	Dead	**10**

2. Oxygenation (if applicable) will be calculated by:
    a. The number of oxygen free days in the first 28 days (to day 29).
    b. The incidence and duration of new oxygen use during the trial, defined as oxygen use that was not present at time of randomization but occurs subsequently

3. Mechanical Ventilation (if applicable):will be calculated using the following
    a. Ventilator free days in the first 28 days (to day 29), defined as 0 if subject dies within 28 days of ventilation, 28-x if successfully liberated after x days, and 0 if ventilated for 28 days
    b. Incidence and duration of new mechanical ventilation use during the trial, defined as mechanical ventilation that was not present at time of randomization but occurs subsequently

4.3.2 Hospitalization

Duration of hospitalization (days)

For Peer Review Only

4.3.3 Mortality

Mortality rates calculated at day 15, 29, and 60 or discharge, whichever comes later.

4.3.4 Safety

The safety of the intervention will be evaluated during the trial period as compared to the control arm as assessed by the cumulative incidence of Grade 3 and above AEs and SAEs using the Division of AIDS (DAIDS) Table for Grading the Severity of Adult and Paediatric Adverse Events, version 2.1 (July 2017).

4.4 Exploratory Outcome

To evaluate the virologic efficacy of, remdesivir or interferonas compared to the control arm as assessed by the percent of subjects with SARS-CoV-2 detectable in OP/NP sample at days 3, 5, 8, 11, 15, and 29, in centres where capable.

Confidential

5 PARTICIPANT SELECTION & WITHDRAWAL

All subjects will be hospitalized with lab confirmed COVID-19. After the screening evaluations have been completed, the investigator or designee is to review the inclusion/exclusion criteria and determine the subject's eligibility for the study.

Only the reason for ineligibility will be collected on screen failures. Subjects who are found to be ineligible will be told the reason for ineligibility if appropriate.

5.1 Inclusion Criteria

Each participant must meet all of the following inclusion criteria to participate in this study:

1. ≥ 18 years of age
2. Has laboratory-confirmed SARS-CoV-2 infection as determined by PCR, or other commercial or public health assay in any specimen prior to randomization.
3. Hospitalized at a participating centre

5.2 Exclusion Criteria

All participants meeting any of the following exclusion criteria at baseline will be excluded from participation in this study:

1. Anticipated transfer to another hospital, within 72 hours, which is not a study site
2. Expected to not survive beyond 24 hours
3. Known allergy to study medication or its components (non-medicinal ingredients)
4. Receiving one of the study drugs at time of enrolment

5.3 Participant Recruitment

It is anticipated that participants with COVID-19 will present to participating hospitals, and that no other efforts to recruit potential subjects are needed.

Patients that are confirmed to have SARS-CoV-2 will be assessed for eligibility.

Screening will begin with a brief discussion with study staff. Subjects may be excluded based on medical history or co-administered medications. Information about the study will be presented to potential subjects (or legally authorized representative) and questions will be asked to determine potential eligibility. Recruitment can occur via verbal consent over a telephone, given the infection control precautions in place for these participants. Deferred consent will also be considered at all relevant locations, under the auspices of local ethics boards.

For Peer Review Only

5.3.1 Randomization Procedures

Subjects will be randomized in a ratio of 1:1:1 to control (standard-of-care) or remdesivir plus standard of care or interferon plus standard of care, using a web-based randomization module managed by WHO, limited to which drugs are available at each participating institution, acknowledging that supply of drug will not be consistent throughout the study.

If additional arms are added to or dropped from the trial, randomization will proceed with an equal probability of assignment to each of the remaining arms. As new interventions are added, the protocol will be amended and reviewed by REBs and applicable regulatory agencies before implementation.

5.3.2 Blinding and Unblinding Procedures

The study will be open-label at the level of patient, research team, and outcome assessment.

5.4 Participant Withdrawal and Discontinuation of IP

Confidential

Participants are free to withdraw from participation in the study at any time upon request, without any consequence. Participants should be listed as having withdrawn consent only when they no longer wish to participate in the study and no longer authorize the Investigators to make efforts to continue to obtain their outcome data.

5.4.1 Reasons for Withdrawal/Discontinuation of IP

Subjects in this clinical trial may discontinue study drug for any of the following reasons:

- Subject requests to discontinue study drug
- Occurrence of any medical condition or circumstance that exposes the patient to substantial risk and/or does not allow the patient to adhere to the requirements of the protocol
- Any serious adverse event (SAE), clinically significant adverse event, severe laboratory abnormality, intercurrent illness, or other medical condition deemed related to the study drug that indicates to the Investigator that continued participation is not in the best interest of the patient
- Patient fails to comply with protocol requirements or study-related procedures

Unless the patient withdraws consent, those who discontinue study drug early should remain in the study for further acquisition of endpoint measurements. The reason for patient discontinuation of study drug should be documented in the case report form.

Should the patient withdraw consent or requests discontinuation for any reason, whenever possible, the patient or substitute decision-maker should be asked if the patient can be followed for outcome and safety evaluations as per protocol. If a patient is enrolled under the deferred consent model, has no available substitute decision-maker, and participant passes away before consent can be sought, data will be recorded for outcome and safety reporting.

For Peer Review Only

5.4.2 Data Collection and Follow-up for Withdrawn Participants

The reason for patient discontinuation from the study will be recorded on the appropriate case report form. In the case of a participants becoming lost to follow-up, attempts to contact the patient should be made and documented in the patient's medical records.

Confidential

For Peer Review Only

6 INTERVENTIONS

6.1 Investigational Product

Remdesivir: Remdesivir is available for dispensing as a lyophilized solid containing 100mg of Remdesivir to be reconstituted.

Interferon-beta-1a: Interferon is available for dispensing as a sterile solution in a prefilled syringe containing 44 or 22 micrograms of drug.

6.1.1 Acquisition, Formulation and Packaging

6.1.1.1 Acquisition and Formulation

Remdesivir

Remdesivir will be provided by WHO. This may change in the near future with changing supply chains, including through hospital supply or direct provision from Gilead or the government of Canada or other.

The lyophilized formulation of Remdesivir is a preservative-free, white to off-white or yellow, lyophilized solid containing 100 mg of Remdesivir to be reconstituted with sterile water for injection and diluted into IV infusion fluids prior to IV infusion. In addition to the active ingredient, the lyophilized formulation of Remdesivir contains the following inactive ingredients: water for injection, SBECD (sulfobutylether cyclodextrin), hydrochloric acid, and/or sodium hydroxide. Hydrochloric acid and/or sodium hydroxide are used to adjust the formulation to a final pH of 3.0 to 4.0 following reconstitution.

Confidential

Interferon

Interferon will be provided by EMD Serono or the WHO. This may change in the near future with changing supply chains, including through hospital supply.

Interferon-beta-1a is supplied as a sterile solution containing no preservative, available in a pre-filled syringe. It is provided as a single-dose prefilled graduated syringe available in two concentrations, either 44 micrograms/0.5 ml or 22 micrograms/0.5 ml. The liquid should be clear to slightly yellow. It contains the following inactive ingredients: mannitol, poloxamer-188, methionine, benzyl alcohol, sodium acetate, water for injection.

6.1.1.2 Packaging

Remdesivir
It is supplied as a sterile product in a single-use, 50 mL, Type 1 clear glass vial.

For Peer Review Only

Interferon
It is supplied as a sterile product in a prefilled syringe.

6.1.2 Treatment Assignment Procedures

All subjects will be randomized to the intervention arms in a 1:1:1 ratio

6.1.3 Dosage, Preparation and Administration

Drug preparation will be performed by the participating site's research pharmacist and/or nurse, as per local procedures, on the same day of administration to the subject

Remdesivir:
Lyophilized solid containing 100 mg of remdesivir will be reconstituted and then diluted into IV infusion fluids (Normal Saline) before use. After reconstitution, the total storage time before administration (including any time before or after dilution) should not exceed 4 hours at room temperature or 24 hours at refrigerated temperature (2°C to 8°C).

Study doses are 200 mg (2 vials) intravenous loading dose on Day 1, and 100mg (1 vial) intravenous once-daily for subsequent doses from Day 2 up to Day 10.

The total volume of administration can be 250 mL or 500 mL of Normal Saline. The infusion can be administered over a period of between 30 minutes and 2 hours.

Interferon
Interferon will be provided as a pre-filled syringe containing 22 or 44 micrograms.44 microgram syringes will be administered to the patient via subcutaneous administration on days 1, 3 and 6.

6.1.3.1 Dosage in special population

Remdesivir: Remdesivir was used in six pregnant women in a RCT testing investigational therapies for Ebola.[2] Although the number of pregnant women was limited, no adverse events were reported in pregnant participants, suggesting the drug to be safe in pregnancy. [2,7] In addition, 16 hospitalized pregnant women with COVID-19 received Remdesivir and no stillbirths were reported.[8] Pregnant women will be informed on the current state of knowledge regarding the safety of Remdesivir in pregnancy so they can make an informed decision regarding trial participation. No dose adjustments will be made for hepatic or renal failure.

Interferon: Safety data is available on Interferon-β1a use in human pregnancy. In a cohort of 679 pregnancies exposed to Interferon-β1a for the treatment of multiple sclerosis for an average of 28 days, the frequency of congenital malformation, stillbirth, and miscarriage was in line with the reported frequency in the general population.[9] Moreover, there was no evidence of increased miscarriage, congenital malformation, low infant birth weight, or impaired

For Peer Review Only

neurodevelopment in over 2,750 exposed pregnancies to Interferons reported in the literature.[10] Pregnant women will be informed on the current state of knowledge regarding the safety of Interferon-β1a in pregnancy so they can make an informed decision regarding trial participation. No dose adjustments will be made for hepatic or renal failure.

6.1.3.2 Notes on Overdose

For management of suspected overdose of any agent, contact local regional Poison Control Centre. There is no specific antidote for overdose. Treatment of overdose should consist of general supportive measures, including monitoring of vitals and observation.

6.1.4 Dose Modification

There are no expected dose modifications.

Missed doses will not be made up.

6.1.5 Accountability: Receiving, Storage, Dispensing and Return

The site PI is responsible for study product distribution and disposition and has ultimate responsibility for study product accountability. The site PI may delegate to the participating site's research pharmacist responsibility for study product accountability.

The participating site's research pharmacist will be responsible for maintaining complete records and documentation of study product receipt, accountability, dispensation, storage conditions, and final disposition of the study product(s).

All study product(s), whether administered or not, must be documented on the appropriate study product accountability record or dispensing log. The sponsor's monitoring staff will verify the participating site's study product accountability records and dispensing logs per the site monitoring plan.

Confidential

6.1.5.1 Receipt of Investigational Product

The study will initiate using products supplied by WHO (Remdesivir) or EMD-Serono (interferon) but as mentioned above, may shift supply depending on availability of products, including using hospital supply where available.

6.1.5.2 Storage and Stability

Remdesivir:

Ambient vials of the lyophilized formulation of Remdesivir should be stored below 30°C. After reconstitution, the total storage time before administration (including any time before or after dilution) should not exceed 4 hours at room temperature or 24 hours at refrigerated temperature (2°C to 8°C).

For Peer Review Only

Interferon:

Interferon is stored at 2-8°C. If needed it can be stored at room temperature up to 25°C, for up to 30 days away from heat and light, but refrigeration is preferred.

6.1.5.3 Dispensing of Investigational Product

Each dose of study product will be administered by a member of the clinical team that is qualified and licensed to administer the study product. Administration and date, time, will be entered into the case report form (CRF) by the research team.

6.1.5.4 Return and/or Destruction of Investigational Product

After the study treatment period has ended or as appropriate over the course of the study after study product accountability has been performed, disposition of unused and used active vials should occur as noted:

Unused and Used active vials:

- Should be destroyed on-site following applicable site procedures or by the site's selected destruction vendor. Following the site's procedure for the destruction of hazardous material or study product destruction policy/standard operating procedure (SOP) when destroying used and unused items.
- A certificate of destruction, if available, should be provided to the sponsor and retained in the Pharmacy Binder once completed.

Confidential

At the completion of the study, there will be a final reconciliation of investigational product shipped, used, and remaining. This reconciliation will be documented. Any discrepancies noted will be investigated, resolved, and documented prior to return or destruction of unused investigational product. Documentation of investigational product destroyed on site and/or off site will be retained in the study files.

6.1.6 Prior and Concomitant Medications/Treatments

Concomitant medications in a hospitalized population change daily and are difficult to collect and attribute to success and failure of therapy and impact on safety. Therefore, only select concomitant medications will be recorded in this trial. The list of medications will be assessed from 7 days prior to enrolment to day 60.

For remdesivir and interferon, there are no known drug interactions.

6.1.7 Safety Considerations

Remdesivir: There are some reports of liver toxicities that have been observed in prior studies. These have been self-limited and resolved after cessation of the medication.

For Peer Review Only

Interferon: There are reports of rare liver toxicities in patients on prolonged use of interferon. Fever and anemia have been reported in the monograph, but were not observed at an appreciable level in the recent randomized clinical trials. [5,6]

For all participants allergies should be assessed by the clinical team and through assessment of medication reconciliation performed on hospital admission.

Confidential

For Peer Review Only

7 STUDY SCHEDULE AND PROCEDURES

Subjects are expected to stay on trial for 60 days or discharge, whichever comes later, but not necessarily hospitalized for the entire period. Below are the study procedures. The Schedule of Assessments (Appendix A) can be referenced at any time.

If a physiologic parameter has abnormal vital signs or laboratory values as per institutional reference ranges, then the measurement may be repeated once if, in the judgment of the investigator, the abnormality is the result of an acute, short-term, rapidly reversible condition or was an error. A physiologic parameter may also be repeated if there is a technical problem with the measurement caused by malfunctioning, or an inappropriate measuring device (i.e., inappropriate-sized BP cuff).

7.1 Screening

After the informed consent, some or all of the following assessments are performed to determine eligibility requirements as specified in the inclusion and exclusion criteria:

- Concomitant Medications (from 7 days prior to enrolment)
- Confirm the positive SARS-CoV-2 test result
- Focused medical history, including the following information:
    - Day of onset of COVID-19 symptoms
    - History of chronic medical conditions related to inclusion and exclusion criteria
    - Medication allergies
    - Review medications and therapies for this current illness and record on the appropriate CRF
- Obtain weight
- Review recent radiographic imaging (x-ray or CT scan)
- Vitals including SpO2

Confidential

The weight and radiographic imaging can be obtained within 7 days prior to the screening visit. The screening process can be suspended prior to complete assessment at any time if exclusions are identified by the study team. Study subjects who meet all eligibility criteria will be immediately randomized.

7.2 Baseline/Enrollment (Study Day 1)

Procedures/Assessments/Evaluations in this section are to be completed after eligibility has been confirmed and the subject has been randomized. These do not need to be performed in the order listed below, but the administration of study medication must be the last procedure to be completed.

- Hospitalization
- Ordinal score
- Oxygen requirement

For Peer Review Only

- Non-invasive mechanical ventilation (via mask)
- Mechanical ventilator requirement (via endotracheal tube or tracheostomy tube)
- ECMO requirement
- Vitals (temperature, pulse, respiratory rate, pulse oximetry, weight, blood pressure and SpO_2)
- Adverse Events since Screening (if screening was not completed on the same day)
- Selected concomitant medication review (if screening was not completed on the same day)
- Oropharyngeal / nasopharyngeal Swabs (if subject is being hospitalized)
- Physical examination: A symptom-directed (targeted) physical examination will be performed by a member of the clinical team in order to evaluate for any possible adverse event.
- Clinical laboratory evaluations (see Table 4: Venipuncture Volumes)– (- 3 Days)
    - Blood will be collected/values recorded according to the time points indicated in the SOE for safety, including ALT, PT/PTT or INR, and creatinine
    - Fasting is not required before collection of laboratory samples.
    - This testing will be performed at each clinical trial site in real time.
- Administration of study medication or control

Table 4: Venipuncture Volumes

Day	1	5 (± 1)
Safety hematology, chemistry and liver tests*	6mL	6mL

*Fasting is not required for any of these lab tests

7.3 Study visits and Follow up

7.3.1 Study Day 2 (+/-1) through Discharge

At each study day while hospitalized, the following procedures will be conducted. Procedures/Assessments/Evaluations do not need to be performed in the order listed below, but the administration of study medication must be the last procedure to be completed:

- Hospitalization
- Ordinal score
- Oxygen requirement
- Non-invasive mechanical ventilation (via mask)
- Mechanical ventilator requirement (via endotracheal tube or tracheostomy tube)
- ECMO requirement
- Collection of available oropharyngeal / nasopharyngeal swab results, if performed.- **Days 3, 5, 8, 11, 15 and 29**
- Clinical laboratory evaluations – **Day 5**
    - Blood will be collected, if not already collected for clinical purposes for safety, including ALT, PT/PTT or INR and creatinine.
    - Fasting is not required before collection of laboratory samples.

- Adverse Events since Screening (if screening was not completed on the same day)

For Peer Review Only

Confidential

- Selected concomitant medication review (if screening was not completed on the same day)
- Administration of study medication or control

7.3.2 Study Days 15 (+/-2), 29 (+/-3) and 60 (+/-3)

The following procedures will be conducted. Procedures/Assessments/Evaluations do not need to be performed in the order listed below. These visits will be conducted by phone if the patient is discharged and the information gathered below will vary.

- Hospitalization
- Ordinal score
- Oxygen requirement
- Non-invasive mechanical ventilation (via mask)
- Mechanical ventilator requirement (via endotracheal tube or tracheostomy tube)
- ECMO requirement
- Adverse events collection – **Days 15 and 29**
- Selected concomitant medication review
- Limitations on activities, if any
- Oropharyngeal / nasopharyngeal swabbing – **Days 15 and 29**

7.3.3 Follow-Up in Pregnant Participants

In pregnant individuals, outcomes will be tracked as following

- If pregnant and deliver in this hospital, information about the delivery and the baby will be captured out to six weeks of age. If they are discharged early, follow-up phone calls will be made to capture this information.
- If they are pregnant and discharged home safely, monthly phone calls will be made until six weeks after delivery, to capture information on pregnancy and baby outcomes. We expect this to be a small number of individuals, and will be captured on a separate case-report form for pregnant individuals, in addition to the main CRF.

7.4 Protocol Deviations

A protocol deviation is any noncompliance with the clinical trial protocol, any process that is noted in the protocol and refers to details in the protocol-specific MOP, or GCP requirements or any critical study procedures with specific instructions in ancillary documents referenced in the protocol such as a protocol-specific MOP.

It is the responsibility of the investigator to ensure that only investigative procedures, as outlined in this protocol are performed on study participants; the occurrence of deviations from the protocol or SOPs are limited; and compliance with the regulations is maintained. Planned deviations from the protocol must not be implemented without prior agreement from the sponsor and approval from the local REB/ethics committee, as required, unless to eliminate an immediate hazard to a participant.

For Peer Review Only

Confidential

Planned or unplanned deviations may occur on the part of the participant, the investigator, or study research team. In resolution to a deviation, corrective/preventative actions are to be developed and implemented in a timely manner. Protocol deviations will be documented and reported as required and assessed where necessary during analysis. All individual protocol deviations will be addressed in subject study records.

It is the responsibility of the site PI and personnel to use continuous vigilance to identify and report deviations within five working days of identification of the protocol deviation, or within five working days of the scheduled protocol-required activity. All deviations must be promptly reported per the protocol deviation reporting procedures. Protocol deviations must be sent to the local REB per their guidelines. The site PI and personnel are responsible for knowing and adhering to their REB requirements. A completed copy of the Protocol Deviation Form must be maintained in the Essential Documents, as well as in the subject's chart if the deviation is subject specific.

Confidential

For Peer Review Only

8 ASSESSMENT OF SAFETY

The safety of research participants is foremost and should always be considered throughout the conduct of research.

For any potential research related injury, the site PI or designee will assess the subject. Study personnel will try to reduce, control, and treat any complications from this study. Immediate medical treatment may be provided by the participating study site. The site PI should then determine if an injury occurred as a direct result of the tests or treatments that are done for this trial.

If it is determined by the participating site PI that an injury occurred to a subject as a direct result of the tests or treatments that are done for this trial, then it shall be recorded as an Adverse Event.

Study personnel will try to reduce, control and treat any complications from this trial. Immediate medical treatment may be provided by the participating site, such as giving emergency medications to stop immediate allergic reactions.

8.1 Definitions

8.1.1 Adverse Events

An adverse event (AE) means any untoward medical occurrence in a patient or clinical investigation participant administered a pharmaceutical product and which does not necessarily have to have a causal relationship with this treatment, and includes an adverse drug reaction (ADR).

An AE can therefore be any unfavorable and unintended sign (including an abnormal laboratory finding, for example), symptom, or disease temporally associated with the use of a medicinal product, whether or not considered related to the medicinal product.

Any medical condition that is present at the time that the subject is screened will be considered as baseline and not reported as an AE. However, if the severity of any pre-existing medical condition increases, it should be recorded as an AE. AEs will be captured from randomization for all study arms.

Given the nature of severity of the underlying illness, subjects will have many symptoms and abnormalities in vitals and laboratory results. All Grade 3 and 4 AEs will be captured as severe AEs in this trial.

The determination of seriousness, severity, and causality will be made by an on-site investigator who is qualified (licensed) to diagnose AE information, provide a medical evaluation of AEs, and classify AEs based upon medical judgment. This includes but is not limited to physicians, physician assistants, and nurse practitioners.

Confidential
For Peer Review Only

All AEs will be assessed for severity, according to the Division of AIDS (DAIDS) Table for Grading the Severity of Adult and Paediatric Adverse Events, version 2.1 (July 2017).

AEs characterized as intermittent require documentation of onset and duration of each episode. The start and stop Duration of each reported AE will be recorded on the appropriate CRF. Changes in the severity of an AE will be documented to allow an assessment of the duration of the event at each level of intensity.

8.1.2 Adverse Events of Special Interest

Liver enzymes that escalate to >5x normal and new dialysis will be collected as adverse events of special interest in this protocol, in all study arms including the control arm. Liver enzymes that escalate to >5x normal will necessitate stopping of the medication in the interventional arms.

If liver enzymes return below that threshold during clinically-obtained bloodwork, local investigators can re-start treatment to complete the allocated duration of administration as per the protocol, up to the original stop date at time of randomization. Missed doses will not be made up.

Confidential

8.1.3 Serious Adverse Events

A serious adverse event (SAE) or reaction is any untoward occurrence that at any dose:

- Results in death
- Is life-threatening,
- Requires inpatient hospitalization or prolongation of existing hospitalization,
- Results in persistent or in significant disability/incapacity,
- is a congenital abnormality or a birth defect.

The term “life-threatening” in the definition of “serious” refers to an event in which the participant was at risk of death at the time of the evet; it does not refer to an event which hypothetically might have caused death if it were more severe.

8.1.4 Unexpected Adverse Event

An unexpected adverse event is any AE that is not identified in nature, severity or frequency in the current Investigator’s Brochure or Product Monograph.

8.1.5 Unexpected Adverse Drug Reaction (ADR)

An ADR is an adverse reaction, the severity of which is not consistent with the applicable Investigator’s Brochure or Product Monograph. All noxious and unintended responses to a medicinal product related to any dose should be considered an ADR.

For Peer Review Only

The phrase “responses to a medicinal product” means that a causal relationship between a medicinal product and an adverse event is at least a reasonable possibility, i.e., the relationship cannot be ruled out.

The expression “causal relationship” is meant to convey that in general there are facts, evidence or arguments to suggest a reasonable causal relationship. All serious and unexpected ADRs will have expedited reporting to regulatory agencies following ICH-GCP and local regulatory requirements.

8.2 Assessment of an Adverse Event

8.2.1 Relationship (Causality/Relatedness)

The causality assessment is the determination, according to the investigator’s clinical judgment, of the existence of a reasonable possibility that the study drug (IP) caused or contributed to an adverse event.

If the investigator or delegated sub-investigator is unsure about whether or not the study drug caused or is related to the event, then the event will be handled as “related” to the study drug for reporting purposes of the trial. If the causality assessment is “unknown but not related” to the study drug, this should be clearly documented in the source documents.

For each reported adverse reaction, the PI or designee must assess the relationship of the event to the study product using the following guideline:

- Unrelated*
- Unlikely related
- Possibly related
- Probably related
- Definitely related**

*There is not a reasonable possibility that the administration of the study intervention caused the event, there is no temporal relationship between the study intervention and event onset, or an alternate etiology has been established.

**The AE is known to occur with the study intervention, there is a reasonable possibility that the study intervention caused the AE, or there is a temporal relationship between the study intervention and event. Reasonable possibility means that there is evidence to suggest a causal relationship between the study intervention and the AE.

8.2.2 Expectedness

Events are classified as unforeseen or unexpected if the nature, severity or frequency is not consistent with the risk information set out in the Product Monograph (PM) or label.

Confidential

For Peer Review Only

8.2.3 Seriousness

Events are classified as serious if associated with effects threatening the life or physiological functions of a participant (SAE). Refer to definition for "Serious Adverse Events" in section 8.1.3.

8.2.4 Severity

The term "severe" is often used to describe the intensity (severity) of a specific event (e.g. mild, moderate, or severe myocardial infarction); the event itself, however, may be of relatively minor medical significance (such as severe headache). This is not the same as "serious," which is based on participant/event outcome or action criteria usually associated with events that pose a threat to a participant's life or functioning. The terms "serious" and "severe" are not synonymous. Seriousness (not severity) serves as a guide for defining regulatory reporting obligations.

For AEs not included in the protocol defined grading system, the following guidelines will be used to describe severity [Division of AIDS (DAIDS) Table for Grading the Severity of Adult and Paediatric Adverse Events, version 2.1 (July 2017)].

- Mild (Grade 1): Events that are usually transient and may require only minimal or no treatment or therapeutic intervention and generally do not interfere with the subject's usual activities of daily living.

- Moderate (Grade 2): Events that are usually alleviated with additional specific therapeutic intervention. The event interferes with usual activities of daily living, causing discomfort but poses no significant or permanent risk of harm to the research subject.

- Severe (Grade 3): Events interrupt usual activities of daily living, or significantly affects clinical status, or may require intensive therapeutic intervention. Severe events are usually incapacitating.

- Severe (Grade 4): Events that are potentially life threatening.

8.3 Adverse Event Recording

Adverse events that are assessed and determined **unrelated or unlikely related** to the investigational product will not be recorded and reported.

In this study,

1. For participants randomized to the investigational product (IP), recording and reporting of adverse events will be restricted to events that are assessed by the site investigator or delegated sub-investigators to be Grade 3 and above and related to the IP (i.e. possibly, probably or definitely related).

Confidential

For Peer Review Only

2. For participants randomized to the standard-of-care arm, severe (Grade 3 and above) adverse events will be recorded and reported, acknowledging the challenges in comparison between the intervention arms. Death will not be collected as an SAE in the standard-of-care arm, since it is collected as an outcome.

Investigations into potential adverse events should be done during each contact with a participant. Investigations may be done through specific questioning and, as appropriate, by examination. Information on all adverse events should be recorded promptly in the source document, and assessed by an investigator or delegated sub-investigators in a timely manner allowing sufficient time to meet required reporting timelines for SAEs and SUADRs if needed. Adverse event CRFs should be completed using source documents by a delegated research team member in a within 15 days for non-SAEs and 96 hours for SAEs of site awareness. All clearly related signs, symptoms, and abnormal diagnostic procedures should be recorded in the source document, though should be grouped under one diagnosis.

If the site investigator or delegated sub-investigator is unsure about whether the event is caused by or related to the investigational product, then the event will be handled as "related" to the study drug for reporting purposes of the trial. If the causality assessment is "unknown but not related" to the investigational product, this should be clearly documented in the source documents.

Confidential

The following are not considered AEs and therefore do not require recording:

- Pre-existing diseases or conditions identified and recorded at screening/baseline unless, at the discretion of the investigator, the disease or condition worsens in severity or frequency
- At the discretion of the investigator, events considered likely manifestations of the underlying disease or that commonly occur in the study population independent of IP exposure
- Elective medical or surgical procedures.

Information on all applicable AEs should be recorded on the appropriate source and in REDCap. All clearly related signs, symptoms, and results of diagnostic procedures performed because of an AE should be grouped together and recorded as a single diagnosis. If the AE is a laboratory abnormality that is part of a clinical condition or syndrome, it should be recorded as the syndrome or diagnosis rather than the individual laboratory abnormality. Each AE will also be described in terms of duration (start and stop date), severity, association with the study product, action(s) taken, and outcome.

8.4 Reporting of SAEs and Unanticipated Events

Serious adverse events and unanticipated events that are assessed and determined not to be related to the investigational product will not be recorded and reported.

8.4.1 Investigator reporting: Notifying the REB

Serious adverse events and unanticipated events should be recorded and reported to the REB in accordance with local reporting requirements and timelines

8.4.2 Investigator reporting: Notifying the Sponsor

The investigator is responsible for reporting serious adverse events and serious and unexpected adverse drug reactions (SUADRs) to the sponsor in accordance with applicable regulations and reporting requirements and timelines.

Events that are assessed to be **serious and unexpected and related or cannot be ruled out as related** to the investigational product are considered SUADRs. Reporting for SUADRs should include a description of the event in sufficient detail to allow for a complete medical assessment of the case and independent determination of possible causality. The minimum information required includes at least one identifiable participant, one identifiable reporter, one serious reaction, and one suspect product.

All SAEs will be forwarded to the DSMB for review if the site PI or appropriate Sub-Investigator becomes aware of an SAE.

8.4.3 Investigator Reporting to Subjects

Subjects will be informed of any severe AEs or SAEs that occur as part of their participation in this trial

8.4.4 Sponsor Reporting of SUADRs: Notifying Health Canada

The regulatory sponsor is responsible for reporting SUADRs to regulatory authorities in accordance with local expedited reporting requirements and timelines. In addition, the Sponsor will complete the ADR Expedited Reporting Summary Form and submit this form to the appropriate Health Canada directorate.

Any unexpected fatal or life-threatening suspected adverse reaction will be reported to the regulatory authority as soon as possible, but in no case later than 7 calendar days after the sponsor's initial receipt of the information. If the event is not fatal or life-threatening, the safety report will be submitted within 15 calendar days after the sponsor determines that the information qualifies for reporting. Relevant follow up information to safety report will be submitted as soon as the information is available. Upon request from regulatory authority, the sponsor will submit any additional data or information that the agency deems necessary, as soon as possible, but in no case later than 15 calendar days after receiving the request.

SAEs that are not SUSARs will be reported to the regulatory authority at least annually in a summary format which includes all SAEs.

Confidential

For Peer Review Only

8.4.5 Sponsor Reporting of SUADRs: Notifying Sites

The regulatory sponsor is responsible for distributing blinded expedited reports of SUADRs to each investigator for submission to local Ethics Committees within 15 days of sponsor awareness.

8.4.6 Sponsor Reporting of AEs: Notifying Abbvie and EMD Serono/Merck KGaA, Darmstadt, Germany.

The regulatory sponsor is also responsible for reporting all relevant and applicable SAEs and AEs to Abbvie and EMD Serono via fax or email within the acceptable calendar days. EMD Serono is a business of Merck KGaA, Darmstadt, Germany.

8.5 Type and Duration of Follow-up for Adverse Events

For this study, all SAEs and Grade 3 and above AEs (using DAIDS 2017) occurring from randomization through Day 29 will be collected. These events recorded during this period will be followed through to resolution, or until the event is assessed as chronic or stable.

8.6 Reporting and Entry Timelines

Study investigators will report SAEs to the sponsor within the following timelines:

- All deaths and immediately life-threatening events deemed to be related to study drug will be recorded and reported to the sponsor within 24 hours of site awareness.
- Serious adverse events other than death and immediately life-threatening events, deemed to be related to study drug will be reported to the sponsor within 72 hours of site awareness.

Adverse event information will be entered into the CRF in a timely manner and **no later than 15 days** from the time the investigator becomes aware of the event.

Serious adverse event information will be entered into the CRF in a timely manner/**within 96 hours** from the time the investigator becomes aware of the event.

8.7 Data and Safety Monitoring Board (DSMB)

Safety oversight will be conducted by a DSMB that is an independent group of experts that monitors subject safety. The DSMB members will be separate and independent of study personnel participating in this trial and should not have scientific, financial or other conflict of interest related to this trial.

Given the unique nature of this trial, with a Canadian sponsor and regulator, and a global coordinating effort, there will be two, distinct DSMBs. The global DSMB will monitor globally accruing data for efficacy integrating the data available. Stopping rules will be determined

Confidential

For Peer Review Only

through the DSMBs, admitting that pre-specified rules for stopping for futility or benefit are difficult to ascertain for a trial of this magnitude.

The Canadian DSMB will consist of three members with appropriate expertise to contribute to the interpretation of the data from this trial. The DSMBs will operate under the guidelines of a charter that will be written at the organizational meeting of the DSMBs. The DSMBs will review SAEs on a regular basis and ad hoc during this trial.

The Canadian DSMB will conduct the following reviews:

- After the first 110 and 220, and then after each 500 subjects.
- Ad hoc meeting if the protocol team or relevant ethics review committees raises any concerns.
- A final review meeting after final clinical database lock in Canada to review the cumulative unblinded safety data for this trial.

The study will not stop enrolment awaiting these Canadian DSMB reviews, though the Canadian DSMB may recommend temporary or permanent cessation of enrolment based on their safety reviews.

Additional data may be requested by the Canadian or Global DSMBs, and interim statistical reports may be generated as deemed necessary and appropriate. The DSMBs may receive data in aggregate and presented by treatment arm. The Canadian DSMB may also be provided with expected and observed rates of the expected AEs in an unblinded fashion and may request the treatment assignment be unblinded for an individual subject if required for safety assessment. The DSMB will review grouped and unblinded data in the closed session only. As an outcome of each review/meeting, the DSMB will make a recommendation as to the advisability of proceeding with study interventions (as applicable), and to continue, modify, or terminate this trial. Clear stopping rules for harm are in sections 10.3.1, and will be integrated into the charter of the DSMB; however, given the adaptive nature of the study, the trial itself will not be stopped, but rather the intervention under study.

Confidential

For Peer Review Only

9 SITE MONITORING, AUDITING AND INSPECTING

9.1 Site Monitoring Plan

Site monitoring is conducted to ensure the safety of human study participants and the protection of their rights and well-being. Monitoring also verifies that collected study data is accurate, complete and verifiable by source documentation and that the study is conducted in accordance with the protocol and operating procedures.

Monitoring for this study is the responsibility of the sponsor. The delegated monitor will evaluate study processes and documentation based on the approved protocol/amendment(s), Part C, Division 5 of the Food and Drug Regulations, the International Council for Harmonisation of Technical Requirements for Pharmaceuticals for Human Use (ICH), E6: Good Clinical Practice guidelines (GCP) and institutional policies. Unique to this circumstance will include virtual monitoring as much as possible – most participating hospitals are restricting access to individuals with recent travel to other hospitals, for risk of contagion.

The extent and nature of monitoring is outlined in the Monitoring Plan. The monitoring plan specifies the frequency of monitoring, monitoring procedures, the level of site monitoring activities (e.g., the percentage of participant data to be reviewed), and the distribution of monitoring reports. Monitoring activities will be performed both in person and remotely. Reports of findings identified during monitoring activities will be provided to sites detailing any required actions. Documentation of monitoring activities and findings will be provided to the site study team and the study QI. The institution and/or local REB reserve the right to conduct independent audits as necessary.

The Investigator is responsible for ensuring monitors and/or quality assurance reviewers are given access to all study-related documents noted above and study related facilities (e.g. pharmacy, diagnostic laboratory, etc.), and have adequate space to conduct the monitoring visit or audit.

9.2 Auditing and Inspecting

The investigator will provide direct access to source data/documents for the purposes of study-related monitoring, audits, and inspections by the REB, the sponsor, and applicable regulatory bodies. The investigator will permit the review of all study related documents (e.g. source documents, regulatory documents, data collection instruments, study data etc.) and will ensure access to applicable study-related facilities (e.g. pharmacy, diagnostic laboratory, etc.).

Confidential

For Peer Review Only

10 STATISTICAL CONSIDERATIONS

This study is intended to allow for adapting to add or subtract experimental arms A brief summary is provided here.

Addition or subtraction of interventions

If additional data become available to add an experimental therapy, analyses of experimental arms will be performed comparing concurrently enrolled control subjects. If one treatment crosses an efficacy stopping boundary, this treatment may become the new control arm for comparisons. This approach was used in the recent PALM Ebola therapy RCT [Mulangu, 2019].

Secondary hypotheses have been ordered according to relative importance. These will be described according to the appropriate summary statistics (e.g., proportions for categorical data, means with 95% confidence intervals for continuous data, median for time-to-event data).

10.1 Study Hypotheses

The primary outcome is hospital mortality, examined across the three allocation arms.

10.2 Sample Size Considerations

This language is taken from the WHO CORE SOLIDARITY protocol:

"No specific sample size is specified in this public health emergency core protocol. Interim results will be kept under review by an independent Global Data Monitoring and Safety Committee, and this Committee will decide how often to conduct interim analyses. It is anticipated that at least several thousand patients will be recruited into this trial. The larger the numbers entered, the more accurate the results will be, but the numbers that can be entered will depend critically on how large the epidemic becomes."

Confidential

10.3 Planned Interim Analyses

Early analyses include monitoring enrolment, baseline characteristics, and follow-up rates throughout the course of the study by the study team.

The Canadian DSMB will monitor ongoing results to ensure patient well-being and safety as well as study integrity. The DSMBs will be asked to recommend early termination or modification only when there is clear and substantial evidence of a treatment difference. More details about the interim analyses are described below as well as a separate guidance document for the DSMB.

10.3.1 Safety Review

Safety analyses will be based a modified intent-to-treat population consisting of all participants who received at least one dose.

For Peer Review Only

Interim safety analyses will occur at approximately 110 and 220, and then at every 500 patients, according to the DSMB recommendations. We believe this is reasonable given that there is accumulating experience globally with these medications in this condition. Safety analyses will evaluate serious AEs by treatment arm and test for differences using a Pocock spending function approach with a one-sided type I error rate of 0.025. This approach is less conservative than what will be used to test for early efficacy results because proving definitive harm of the experimental agents is not the focus of this study. Pocock stopping boundaries at the looks described correspond to z-scores of (2.37, 2.37, 2.36, & 2.35). This contrasts with the z-score stopping boundaries for the Lan-DeMets spending function that mimics O'Brien-Fleming boundaries: (4.33, 2.96, 2.36 & 2.01). The unblinded statistical team will prepare these reports for review by the Canadian DSMB.

The protocol team will review blinded pools of AE data every 2 weeks to ensure there are no significant number of unexpected AEs (AEs that do not fit with the known course of COVID-19, based on emerging data from the published and unpublished literature). If there are a significant number of unexpected AEs, the DSMB will be asked to review unblinded safety data in an ad hoc meeting.

10.3.2 Efficacy Review

Efficacy reviews will be under the jurisdiction of the global DSMB, with accruing data globally. As outlined earlier, there are no pre-specified stopping rules in place.

10.4 Stopping Rules

If there are a concerning number of unexpected AEs, the DSMB will be asked to review unblinded safety data in an ad hoc meeting. This study may be prematurely terminated if there is sufficient reasonable cause, including but not limited to:

- Determination of unexpected, significant, or unacceptable risk to subjects
- Results of interim analysis
- Insufficient compliance to protocol requirements
- Data that are not sufficiently complete and/or not evaluable
- Regulatory authorities

Mathematical stopping rules are outlined in section 10.3.1

If the study is prematurely terminated, the site QI will promptly inform study subjects and the REB as applicable. The site QI will assure appropriate follow-up for the subjects, as necessary. The global DSMB will be notified accordingly.

The sponsor will notify regulatory authorities as applicable

10.5 Final Analysis Plan

The primary analysis will be based on an intention-to-treat population, including participants randomized and consented.

For Peer Review Only

10.5.1 Primary Efficacy Endpoint

Hospital mortality will be used to estimate the efficacy of specific intervention arms. Specific thresholds for superiority and significance are not specified at this point.

Efforts to minimize loss-to-follow-up will be considerable. However, small amounts of missing data may occur. In such cases, participants without final outcome data of hospital mortality will be excluded from the analysis. Sensitivity analyses will evaluate the impact of making different assumptions about missing observations. These sensitivity analyses will be fully defined in the SAP.

10.5.2 Secondary Endpoint

1) Differences in time-to-event endpoints (e.g., time to a one category improvement in ordinal scale) by treatment will be summarized with Kaplan-Meier curves and 95% confidence bounds.
2) Change in ordinal scale at specific time points will be summarized by proportions (e.g., proportion who have a 1-, 2-, 3-, or 4-point improvement or 1-, 2-, 3-, 4-point worsening).
3) Duration of event (e.g., duration of mechanical ventilation) will be summarized according to median days with quartiles.
4) Incidence data (e.g., incidence of new oxygen use) will be summarized as a percent with 95% confidence intervals.
5) Categorical data (e.g., 29-day mortality or ordinal scale by day) will be summarized according to proportions with confidence intervals on the difference or odds ratios for a binary or multiple category scale, respectively.

Missing data procedures will be described in the SAP.

confidential

10.5.3 Safety Analysis

Safety endpoints include death through Day 60, SAEs, and severe AEs. These events will be analyzed univariately and as a composite endpoint. Time-to-event methods will be used for death and the composite endpoint. Each AE will be counted once for a given participant and graded by severity and relationship to COVID-19 or study intervention. AEs will be presented by system organ class, duration (in days), start- and stop-date. Adverse events leading to premature discontinuation from the study intervention and serious treatment-emergent AEs should be presented either in a table or a listing.

10.5.4 Sub-Group Analysis

Subgroup analyses for the primary outcomes will evaluate the treatment effect across the following subgroups: duration of symptoms prior to enrolment, age <55, sex, and severity of symptoms on presentation (defined as need for positive pressure ventilation, including high-flow nasal cannulae and non-invasive ventilation). A forest plot will display confidence intervals across subgroups. Interaction tests will be conducted to determine whether the effect of treatment varies by subgroup

For Peer Review Only

10.5.5 Exploratory Analysis

An exploratory analysis will compare treatment efficacy estimates according to the various scales outlined in section 3.3.

Confidential

For Peer Review Only

11 DATA HANDLING AND RECORD KEEPING

11.1 Confidentiality

Information about study participants will be kept confidential and managed according to the requirements of the Personal Health Information Protection Act of 2004 (PHIPA) and the Research Ethics Board. As part of the study, no PHI will be collected onto the central databases. Given the unique nature of the study and the unique data flows, these will be outlined below.

All study data that leave the site (including any electronic transmission of data) will be identified only by a coded number that is linked to a subject through a code key maintained at the clinical site.

11.2 Source Documents

Confidential

Source data/documents are original documents, data and records in a clinical study that are necessary for the reconstruction and evaluation of the study. Examples of these original documents and data records include, but are not limited to:

- Worksheets
- hospital records
- medical records
- memorandum
- participants' diaries or evaluation checklists
- pharmacy dispensing records
- copies or transcriptions certified after verification as being accurate and complete
- participant files and records kept at the pharmacy
- entries entered directly into the printed CRF

Each participating site will maintain appropriate medical and research records for this study, in addition to regulatory and institutional requirements for the protection of confidentiality of participants. If electronic source data documents are printed it should be signed and dated by the investigator to confirm content and filed with other source documents.

The investigator(s) and research team members listed on the Task Delegation Log (TDL) will have access to participant medical records and will collect only the information needed for the study. Sponsor delegated monitors, representatives of institutional committees and regulatory authority representatives of the country in which the study is being conducted will also have access to examine records for the purposes of quality assurance reviews, audits and evaluation of study safety and progress.

For Peer Review Only

Source data should be attributable, legible, contemporaneous, original, accurate, and complete. Each participating site will maintain appropriate medical and research records for this trial, in compliance with ICH GCP, regulatory, and institutional requirements. Data recorded in the CRF derived from source documents should be consistent with the data recorded on the source documents.

Interview of subjects is sufficient for obtaining medical history. Solicitation of medical records from the subject's primary care provider is not required.

11.3 Data Management Responsibilities

Data collection and accurate documentation are the responsibility of the study personnel under the supervision of the investigator. All source documents and applicable laboratory reports should be reviewed as needed and used to ensure that data collected for the purposes of the study are accurate and complete. Contemporaneous review of laboratory results and the assessment of clinical significance for those results considered out of range should be documented by means of dated signature by the reviewing investigator. Study personnel, including data entry team members, should use source documents to complete case report forms (CRFs).

As part of the safety plan for this study, the investigator will review individual study participant records to ensure that appropriate mechanisms to protect the safety of study participants are being followed, that protocol requirements are being adhered to, and that data is accurate, complete, and secure. Participant records include, but are not limited to: consent forms, case report forms, data forms, laboratory specimen records, inclusion/exclusion forms, and medical charts. All study data will be collected by a member of the study research team and recorded in accordance with applicable procedures.

Clinical research data from source documentation (including, but not limited to, AE/SAEs, concomitant medications, medical history, physical assessments, clinical laboratory data) will be entered by the clinical study site into CRFs via a secure REDCAP server based at Sunnybrook Research institute The data system includes password protection and internal quality checks, such as automatic range checks, to identify data that appear inconsistent, incomplete, or inaccurate.

The sponsor is responsible for review of data collection tools and processes, and review of data and reports.

11.4 Data Capture

This is a unique study infrastructure, described below. As part of the global WHO SOLIDARITY protocol, randomization will be under the auspices of a central server managed by WHO. It will be a web-based module). Into this database will be entered the following variables:

- Country, hospital, randomizing individual
- Admission date, patient age, patient sex

Confidential

For Peer Review Only

- Co-morbidities: smoking, diabetes, heart disease, chronic lung disease, chronic liver disease, asthma, HIV, active tuberculosis
- Severity of disease: shortness of breath, on oxygen, on ventilator, CXR findings
- Name of study drug given and duration of treatment
- Duration of intensive care/duration of ventilation
- Date of discharge, with associated outcome (dead/alive)
- Cause of death
- SUSARs

The Canadian database will be entered all of the above, and everything else on the CRF to possess a more granular description of the patient population for Canadian interpretation of the collected dataset, via a REDCAP CRF (see below). Included in this database will be allocation status.

11.4.1 Case Report Forms

The study case report forms (CRF) are the primary data collection instrument for the study. Electronic/Paper case report forms (eCRFs/pCRFs) will be used to collect data for this study, with electronic at the WHO module and paper or electronic for the Canadian REDCAP. CRFs are to be completed by data capture personnel and signed off by the investigator in a timely manner. Good documentation practices should be implemented according to standard operating procedures. All data requested on the CRF must be recorded and verifiable by source document.

11.5 Records Retention

It is the responsibility of the REB, investigator and regulatory sponsor to retain study essential documents as per local regulatory requirements and GCP Guidelines.

Study essential documents must be maintained in a secure and confidential manner for participating Canadian sites for a period of 25 years. For the purposes of this study, the start date of the retention period is the date of the final report of the trial. Exceptions may be made for sites which close prematurely, wherein the start date for the retention period will be the date of notification to Health Canada of the sites closure. All study records are then to be destroyed according to local and national policy and requirements. It is the investigator's responsibility to request authorization for destruction at the completion of the retention period and/or for the sponsor to inform the investigator/institution when these documents may be destroyed.

Confidential

11.6 Clinical Trial Registration

In accordance with Health Canada's Notice "Registration and Disclosure of Clinical Trial Information, November 30, 2007", the sponsor will be responsible for registering the study on Clinicaltrials.gov (*www.clinicaltrials.gov*), a publically available registry that conforms to international standards for registries.

Confidential

For Peer Review Only

12 QUALITY CONTROL AND QUALITY ASSURANCE

As per ICH-GCP and local regulations, the Canadian sponsor is responsible for ensuring the implementation and maintenance of systems that support quality assurance and quality control.

The study must be conducted in compliance with the study protocol and all data collected must be accurate and verifiable by source document(s). For the purpose of monitoring and auditing by the Sponsor, and inspection by regulatory authorities, the site will provide direct access to all study related source data/documents. The sponsor will verify that the study is conducted and data has been collected, documented (recorded), and reported in compliance with the protocol, GCP, and applicable regulatory requirements.

Data for the CATCO study will be centrally stored and managed by the Centre for Clinical Trial Support (CCTS) and the data for the WHO-SOLIDARITY study will be under the auspices of the WHO. To ensure the quality of study data, quality assurance and control systems will be implemented using validated electronic quality control checks within the REDCAP electronic data capture system. These verification measures will identify missing data, inconsistencies and/or data anomalies. Both electronic and manual queries will be generated for resolution and review by sites.

Access to secure and validated electronic systems used for the purposes of this study will be controlled by the sponsor. Access will only be granted to individual research team members upon review of training and qualification and authorization by delegation of the investigator.

Quality assurance and control measures will be implemented to ensure training for specific trial–related tasks beyond the usual scope of practice.

Confidential

For Peer Review Only

13 ETHICS CONSIDERATIONS

13.1 Ethical Standard

The investigator will ensure that this study is conducted in accordance with the principles set forth in The Belmont Report: Ethical Principles and Guidelines for the Protection of Human Participants of Research, and codified in the Tri-Council Policy Statement and/or the ICH E6.

13.2 Research Ethics Board (REB)

The protocol, informed consent form(s), recruitment materials, and all participant materials will be submitted to the REB for review and approval. Approval of both the protocol and the consent form must be obtained before any participant is enrolled. Any amendment to the protocol will require review and approval by the REB before the changes are implemented in the study, unless to eliminate an immediate hazard.

REB review and approval will occur at least annually throughout the duration of the study. The investigator will notify the REB of deviations from the protocol and SAEs, as applicable to the REB policy

Confidential

13.3 Consent

Informed consent is a process that is initiated prior to the individual agreeing to participate in the study and continues throughout study participation. As an alternative to written consent, verbal consent will be sought from the patient through a scripted discussion or complete reading of the Informed Consent Form describing in detail the study procedures and risks and answer any questions they might have. This will be witnessed by another healthcare provider or research staff. If verbal consent was given, the care team will document this on a modified consent form which can be given to the patient during hospitalization, to be signed and returned to the site sometime before the end of their participation in the trial. The modified consent forms or scripts will be REB-approved. Additional instruction on the procedures, types of witnesses and other details pertaining to obtaining and documenting informed consent will be outlined in a Manual of Operations. In the case where the participant requires a legal representative, an REB-approved Consent Form for a substitute decision maker will be signed prior to any study-related assessments or procedures are conducted. Participants will be given the opportunity to think about it prior to agreeing to participate, and may request that their surrogate be aware of the study risks as well. They may withdraw consent at any time throughout the course of the study.

Prior to involvement in any study-related activities, consent must be obtained verbally for each participant using the current REB approved informed consent form, or through their deferred consenting process. It is the responsibility of the investigator to ensure that all advertisements and written information, including the informed consent form, disseminated to participants has been approved by the local REB prior to use. Neither the investigator nor study staff should

For Peer Review Only

unduly influence or coerce a participant to participate in the study. Deferred consent will be pursued at a number of sites. This will consist of early randomization, assignment to relevant study arm, with deferred consent sought after from the participant, when the participant is capable or substitute decision maker is available. All efforts must be made from the study team to obtain deferred consent. Pregnant patients should not be enrolled via deferred consent method, given limited safety data on remdesivir and interferon-beta-1a during pregnancy, full informed consent should be obtained from these patients.

The ICF will be signed and dated by the individual obtaining consent and a witness. The consent process will be documented in the clinical or research record.

The original ICF, in its entirety, will be maintained by the site, and a complete copy of the signed ICF provided to the participant at the time of discharge. The rights and welfare of the participants will be protected by emphasizing to them that the quality of their clinical care will not be adversely affected if they decline to participate in this study.

The provision of consent is an ongoing process and should be maintained throughout the duration of the study. Participants may withdraw consent at any time throughout the course of the study.

13.4 Secondary Use of Stored Data

Secondary Human Subject Research is the re-use of de-identifiable data or de-identified bio specimens that were collected from some other "primary" or "initial" activity, such as the data collected in this protocol. Any use of the data for secondary research purposes, however, will be presented in a separate protocol and require separate REB approval.

The subject's decision can be changed at any time by notifying the study doctors or nurses in writing. If the subject subsequently changes his/her decision, the samples will be destroyed if the samples have not been used for research or released for a specific research project. Fully anonymized data that has been entered into the global WHO will be stored for future analysis

Confidential

For Peer Review Only

14 PUBLICATION/DATA SHARING POLICY

As a global trial conducted under the auspices of the World Health Organization, publications will be through that primary analysis at the global level. Following completion of the study, the international collaboration will be disseminated rapidly by the World Health Organization under a group authorship. As the global sponsor for this work, the World Health Organization will store the relevant data collected.

14.1 Data Sharing

To avoid premature release of data, this protocol specifies that efficacy data from a trial that has not yet been completed due to insufficient enrolment should not be released. After an outbreak has ended at a given site, the study would be paused.

A DSMB committee would review results from an interim analysis of study data to make recommendations regarding whether the study should continue or stop for efficacy, futility, or safety, guided by the pre-specified monitoring plan.

As per World Health Organization policies on data sharing in a Public Health Emergency, any clinical trial outcome data will be shared at the earliest possible opportunity. In addition, given the nature of this protocol, being performed across regions, the DSMB may access other regions trials, and possibly recommend alterations in study design based on accumulating data, through a centralized data repository being built under the auspices of the World Health Organization.

Confidential

14.1.1 Data Sharing for Secondary Research

Data from this study may be used for secondary research. All of the individual subject data collected during the trial will be made available after de-identification through expert determination. The SAP and Analytic Code will also be made available. This data will be available immediately following publication, with no end date, as part of data sharing requirements from journals and funding agencies.

14.2 Conflict of Interest

The independence of this study from any actual or perceived influence, such as by the pharmaceutical industry, is critical. Therefore, any actual conflict of interest of persons who have a role in the design, conduct, analysis, publication, or any aspect of this trial will be disclosed and managed. Furthermore, persons who have a perceived conflict of interest will be required to have such conflicts managed in a way that is appropriate to their participation in the design and conduct of this trial.

For Peer Review Only

15 LITERATURE REFERENCES

1. Chan JF, Yuan S, Kok KH, et al. A familial cluster of pneumonia associated with the 2019 novel coronavirus indicating person-to-person transmission: a study of a family cluster. *Lancet* 2020; **395**(10223): 514-23.

2. Mulangu S, Dodd LE, Davey RT, Jr., et al. A Randomized, Controlled Trial of Ebola Virus Disease Therapeutics. *N Engl J Med* 2019; **381**(24): 2293-303.

3. Wang Y, Zhang D, Du G, et al. Remdesivir in adults with severe COVID-19: a randomised, double-blind, placebo-controlled, multicentre trial. *Lancet* 2020; **395**(10236): 1569-78.

4. Beigel JH, Tomashek KM, Dodd LE, et al. Remdesivir for the Treatment of Covid-19 - Preliminary Report. *N Engl J Med* 2020.

5. Hung IF, Lung KC, Tso EY, et al. Triple combination of interferon beta-1b, lopinavir-ritonavir, and ribavirin in the treatment of patients admitted to hospital with COVID-19: an open-label, randomised, phase 2 trial. *Lancet* 2020.

6. Ranieri VM, Pettila V, Karvonen MK, et al. Effect of Intravenous Interferon beta-1a on Death and Days Free From Mechanical Ventilation Among Patients With Moderate to Severe Acute Respiratory Distress Syndrome: A Randomized Clinical Trial. *JAMA* 2020.

7. Dashraath P, Wong JLJ, Lim MXK, et al. Coronavirus disease 2019 (COVID-19) pandemic and pregnancy. *Am J Obstet Gynecol* 2020; **222**(6): 521-31.

8. Pierce-Williams RAM, Burd J, Felder L, et al. Clinical course of severe and critical COVID-19 in hospitalized pregnancies: a US cohort study. *Am J Obstet Gynecol MFM* 2020: 100134.

9. Sandberg-Wollheim M, Alteri E, Moraga MS, Kornmann G. Pregnancy outcomes in multiple sclerosis following subcutaneous interferon beta-1a therapy. *Mult Scler* 2011; **17**(4): 423-30.

10. Medications used to treat COVID-19 in pregnancy. *https://www.medicinesinpregnancy.org/bumps/monographs/MEDICATIONS-USED-TO-TREAT-COVID-19-IN-PREGNANCY/*. Published 2020. Accessed May 6th, 2020.

11. Niemeyer D, Zillinger T Muth D et a;., Middle East Respiratory Syndrome Coronavirus Accessory Protein 4a Is a Type I Interferon Antagonist Journal of Virology Oct 2013, 87 (22) 12489-12495;

12. Hart bj, dyall j, postnikova, e. Interferon-β and mycophenolic acid are potent inhibitors of middle east respiratory syndrome coronavirus in cell-based assays. J Gen Virol 2014 mar 95:571-77

For Peer Review Only

13. Kiss J et al., IFN-beta protects from vascular leakage via up-regulation of CD73. European Journal of Immunology. 2007 37(12); 3334-3338

Confidential

For Peer Review Only

APPENDIX A: SCHEDULE OF EVENTS

	Screening	Baseline	Daily until hospital discharge	Follow-Up		
**Day (+/- Window)**	**0 or 1**	**1**	**1(± 1)**	**15[6] ± 2**	**29[6] ± 3**	**60[6] ± 3**
**Assessments/Procedures**						
**ELIGIBILTY**						
Informed consent	X					
Demographics & Medical History	X					
Review SARS-CoV-2 results	X					
**STUDY INTERVENTION**						
Randomization		X				
Administration of Control/Remdesivir/Interferon/ Remdesivir + Interferon		Daily administration until D6/D10/discharge				
**STUDY PROCEDURES**						
Vital signs including SpO_2	X	X[5]	Daily until discharge	X	X	
Review recent radiographic imaging	X					
Selected Concomitant medication review	X	X[5]	Daily until discharge	X	X	X
Clinical data collection[1]		X[5]	Daily until discharge	X	X	X
Adverse event evaluation		X	Daily until discharge	X	X	
**SAFETY LABORATORY**						
Safety haematology, chemistry and liver tests[2,4]		X[3,5]	Day 5,			
**RESEARCH LABORATORY, EXPLORATORY**						
Oropharyngeal/ nasopharyngeal swab (optional)		X[5,7]	Day 1, 3, 5, 8, 11 (all ± 1 day) if hospitalized[7,]	X[7]	X[7]	

Notes:

1. *Refer to Section 7.3 of the protocol for details of clinical data to be collected. This includes ordinal score, oxygen requirement, Mechanical ventilator requirement etc.*
2. *ALT, PT/PTT or INR, and creatinine.*
3. *Laboratory tests performed in the 72 hours prior to enrolment will be accepted at baseline*
4. *Any laboratory tests performed as part of routine clinical care within the specified visit window can be used for safety laboratory testing.*
5. *Baseline assessments should be performed prior to study drug administration*
6. *These visits will be conducted by phone.*
7. *The swab will be done as per standard of care, and available results will be collected.*

Confidential

For Peer Review Only
