# Supplementary material for: Remdesivir in Patients With Severe Kidney Dysfunction: A Secondary Analysis of the CATCO Randomized Trial
Source: JAMA Netw Open. 2022 Aug 29;5(8):e2229236. doi: 10.1001/jamanetworkopen.2022.29236 (PMC9425145; doi:10.1001/jamanetworkopen.2022.29236)
Supplement: Supplement 3. — Data Sharing Statement [file jamanetwopen-e2229236-s003.pdf]

Data Sharing Statement

Cheng. Remdesivir in Patients With Severe Kidney Dysfunction. *JAMA Netw Open*. Published August 29, 2022. doi:10.1001/jamanetworkopen.2022.29236

Data
Data available: Yes
Data types: Deidentified participant data, Data dictionary
How to access data: catco@sunnybrook.ca
When available: beginning date: 01-01-2023

Supporting Documents
Document types: None

Additional Information
Who can access the data: Researchers whose proposed use of the data has been approved
Types of analyses: For any purpose
Mechanisms of data availability: Without investigator support, will be available on clinical trial data repository
